# Protective effect of *Pistacia lentiscus *L. gum on pentylenetetrazol-induced seizures: Evaluation of antioxidant capacity in the hippocampus of male rats

**DOI:** 10.22038/ajp.2025.25774

**Published:** 2025

**Authors:** Hossein Hassani Kordkolai, Mehdi Sadegh, Maryam Nazari, Masoumeh Gholami

**Affiliations:** 1 *Department of Physiology, School of Medicine, Arak University of Medical Sciences, Arak, Iran*

**Keywords:** Seizure, Pistacia lentiscus L., Pentylenetetrazol, Oxidative stress

## Abstract

**Objective:**

*Pistacia lentiscus *L. (Mastaki), from the Anacardiaceae family, is known for its polyphenolic properties. Regarding its role in oxidative stress, we aimed to investigate its beneficial effects on pentylenetetrazol (PTZ) model of acute seizure.

**Materials and Methods:**

Acute tonic-clonic seizures were induced by PTZ (80 mg/kg; i.p.), with seizure scores assessment done within 30 min using the Racine scales. In the current study, a total of 70 male Wistar rats were randomly added into seven groups of 10: (1) Control, (2) PTZ (80 mg/kg), (3) Vehicle (distilled water + PTZ), (4-6) *P. lentiscus *L. gum groups + PTZ, and (7) Diazepam (0.3 mg/kg) + PTZ. Groups 3-6 received oral (gavage) distilled water or *P. lentiscus *L. gum (100, 200, or 400 mg/kg), 40 min before a single intraperitoneal injection of PTZ. Finally, hippocampal tissues were assessed for the determination of oxidative stress parameters.

**Results:**

*P. lentiscus* gum (400 mg/kg) significantly increased latencies to the stages 2, 4, and 5 of seizure while reduced stage 5 duration in comparison with the PTZ group. Treatment with *P. lentiscus* also decreased elevated malondialdehyde (MDA) levels induced by PTZ and increased glutathione (GSH) concentration, and superoxide dismutase (SOD) and catalase (CAT) activities in comparison with the PTZ group.

**Conclusion:**

This study’s findings indicate that *P. lentiscus *L. possesses anticonvulsant properties, which may be partially attributed to its antioxidant activity, offering protection against oxidative stress.

## Introduction

Epilepsy as a neurological disorder affects one percent of human population (Mehrabani et al. 2007). Although many studies have been carried out to uncover the pathogenic mechanisms of epilepsy, many aspects remain unknown (Sendrowski and Sobaniec 2013). Studies have demonstrated that recurrent and prolonged seizures can contribute to cognitive and emotional disorders (Kiasalari et al. 2013), deteriorate patients’ quality of life, increase the risk of brain damage, and may even lead to mortality (Jalili et al. 2014). Moreover, uncontrolled epilepsy not only imposes significant limitations on daily activities but also inflicts damage to brain cells, underscoring the critical importance of managing and treating this condition (Bhutada et al. 2010). The utilization of anti-epileptic medications is consistently linked to a multitude of side effects, with drug resistance reported in around 30% of patients with epilepsy or epileptic patients (Zareie et al. 2018). Anti-epileptic drugs are not suitable for long-term use due to side effects like teratogenicity. On the other hand, the inability to control convulsive episodes can significantly disrupt an individual’s quality of life (Shahriari et al. 2004). Consequently, the exploration of novel antiepileptic drugs is a crucial endeavor.

Many studies have investigated the therapeutic benefits of medicinal plants for a range of diseases including epilepsy, showing promising results in different animal models (Porter and Meldrum 2001). *Pistacia lentiscus *L., Mastaki or mastic, belonging to the Anacardiaceae family, is a native plant found in regions with a Mediterranean climate, North Africa, and the Greek islands. The *Pistacia* genus comprises approximately 20 species, among which are well-documented varieties such as the pistachio (*Pistacia vera*), the mastic tree (*Pistacia lentiscus*), as well as *Pistacia atlantica*, *Pistacia terebinthus*, and others (Rauf et al. 2017). In the studies that were carried out, antioxidant, anti-atherogenic, anti-cancer, anti-gout, anti-arthritic, anti-inflammatory and wound-healing effects of mastaki have been highlighted. Additionally, research has indicated that certain *Pistacia* species exhibit notable activity within the central nervous system (CNS). For instance, extracts of *P. integerrima* and *P. vera* have demonstrated anticonvulsant properties in model of pentylenetetrazol (PTZ) -induced seizures (Balan et al. 2007). Moreover, *P. lentiscus* has been traditionally used as a treatment for epilepsy; however, there exists a lack of empirical research substantiating its effectiveness and elucidating its mechanism of action in the context of epileptic disorders (Fatehi et al. 2017). According to the studies, the antioxidant characteristics of *P. lentiscus* appear to be a crucial factor in potentially mitigating the effects induced by seizures, as the involvement of oxidative stress in the pathophysiology of epilepsy is well-established. Findings from animal studies suggest an elevation in lipid peroxidation (LPO) during PTZ-induced seizures, concomitant with a decline in the activity of antioxidant enzymes (White 1999). Certain studies have shown that antioxidants such as ascorbic acid and alpha-tocopherol have protective effects against seizures (Aguiar et al. 2012). Additionally, several studies have firmly established the substantial antioxidant activity of *P. lentiscus*, comparable to that of synthetic antioxidants. A previous study demonstrated that *P. lentiscus* notably improved oxidative status (Hosseinzadeh et al. 2012). Consequently, it is conceivable that *P. lentiscus* may diminish the severity of seizures by inhibiting oxidative stress. 

Consequently, the current study sought to evaluate the effects of *Pistacia lentiscus* L. plant gum on PTZ-induced seizures in association with biochemical parameters in the hippocampus of male rats.

## Materials and Methods

### Animal grouping and treatment

In this study, a total of 70 male Wistar rats, each weighing approximately 170 ± 30 g, were used. The rats were kept in an animal facility with controlled conditions, which included a 12-hr light-dark cycle, a temperature maintained between 22-24°C, and suitable humidity levels. They had unrestricted access to standard laboratory food and water. The subjects were randomly placed into seven groups of 10, and received the following treatments.

- Control (Saline)

- PTZ (80 mg/kg)

-Vehicle (distilled water) + PTZ

- *Pistacia lentiscus *L*.* gum 100 mg/kg + PTZ

*- Pistacia lentiscus *L. gum 200 mg/kg + PTZ

*- Pistacia lentiscus *L. gum 400 mg/kg + PTZ

- Diazepam (0.3 mg/kg) (Lahat et al. 2000) + PTZ

### Plant materials

*P. lentiscus *L. gum and leaves were collected from the Dena foothills of Iran (2022 October). Leaves were used to verify plant species at the herbarium department of Arak University (Arak, Iran) and the gums were separated, cleaned, and dried at room temperature, and then powdered and used. A voucher specimen (No. 11200) of the plant was deposited at the Traditional and Complementary Medicine Research Center of the Arak University of Medical Sciences Arak, Iran. The powdered gum was dissolved in a solvent (distilled water). Gavage administration was carried out in groups 3, 4, 5, and 6, 40 min prior to the PTZ injection. The volume for both the injection and the gavage in all animals was standardized at 1 ml.

**Table 1:** Modified Racine’s Scale

**Score**	**Behavioral Expression**
0	Absence of behavioral signs
1	Ear and facial twitching
2	Head nodding and myoclonic jerks
3	Clonus in one forelimb accompanied by a lordotic posture
4	Bilateral forelimb clonus accompanied by rearing and collapsing
5	Generalized tonic–clonic seizure with loss of postural tone

### PTZ-induced tonic-clonic seizures

Acute tonic-clonic seizures were induced in rats via intraperitoneal (i.p.) administration of PTZ at a dosage of 80 mg/kg (Gholami et al. 2020). After the injection, the rats were placed in a clear plexiglass box measuring 40 × 40 × 40 cm, allowing for unobstructed visibility of the animals, and their behavioral seizures were documented for 30 min using a blind assessment technique. The observed convulsive behaviors were classified according to stages 0-5 of the Racine scale, with the latency times of stages 2,4 and 5 reported and stage 5 durations (Hosseini et al. 2020; Racine 1972) (Table 1). 

### Biochemical assay

After finishing the seizure scores recording, animals were deeply anesthetized with urethane injection (1.5 g/kg; i.p.) (Hosseini et al. 2015). The hippocampal tissues were collected and stored at −80°C for later processing. Samples were homogenized in 1000 µl of phosphate buffer solution and centrifuged at 1500 rpm for 10 min. Supernatants were used for measurement of malondialdehyde (MDA), glutathione (GSH), and the activities of superoxide dismutase (SOD) and catalase (CAT).

### MDA level measurement

Malondialdehyde (MDA) is known as a lipid peroxidation biomarker. The assessment method has been described in previous studies (Beheshti et al. 2017). In brief, 1 ml of each sample was added into a solution of 2 ml of thiobarbituric acid and trichloroacetic acid (TCA + hydrochloric acid) boiled for 45 min. The final solution was centrifuged at 1000 g for 10 min, and its absorbance was detected at 535 nm with Epoch 2 Microplate Spectrophotometer, Biotek, USA (Moghimian et al. 2023).

### Glutathione (GSH) measurement 

The assay was conducted in accordance with the methodology delineated in prior research by Rahman et al. Briefly, the interaction between glutathione (GSH) and 5,5'-dithiobis (2-nitrobenzoic acid) (DTNB) results in the formation of the TNB chromophore which exhibits peak absorbance at 412 nm, alongside the oxidized glutathione –TNB adduct (GS–TNB). The TNB formation rate, as measured at 412 nm, is directly proportional to the concentration of GSH present within the sample (Rahman et al. 2006).

### Superoxide dismutase activity

To assess superoxide dismutase (SOD) activity, we followed the established protocol by Madesh and Balasubramanian (1997). It is based on the auto-oxidation of pyrogallol, and generating SOD which results in the inhibition of the reduction of 3-(4,5-dimethyl-thiazol-2-yl) 2,5-diphenyl tetrazolium bromide (MTT) to formazan. Then, dimethyl sulfoxide (DMSO) was added to terminate the reaction. Practically, supernatant from the samples were placed into the wells of a 96-well plate. After a 5-min incubation, DMSO was introduced, and the plate was analyzed using a microplate reader at a wavelength of 570 nm. One unit of SOD is defined as the amount of protein needed to inhibit 50% of the reduction of MTT (Madesh and Balasubramanian 1997).

### Catalase activity

CAT activity was assessed as follows: 100 µl of H2O2 and phosphate buffer (pH 7) were mixed and used to prepare a solution designated for measurement (C buffer). Phosphate buffer (650 µl; pH 7) was used as a blank. The C buffer and homogenized samples were placed into the measurement cuvette. Decreased absorption over a period of 5 min was determined using a spectrophotometer at a wavelength of 240 nm (Aebi 1984).

### Data Analysis

Data are presented as mean±SEM. Statistical software GraphPad Prism 6.0 was used. To assess statistically significant differences among groups of more than two, one-way ANOVA and Tukey’s post-test was conducted. Significance was considered when p<0.05.

## Results

### The effect of P. lentiscus L. gum on the latency and duration of seizure stages

**Figure 1:**
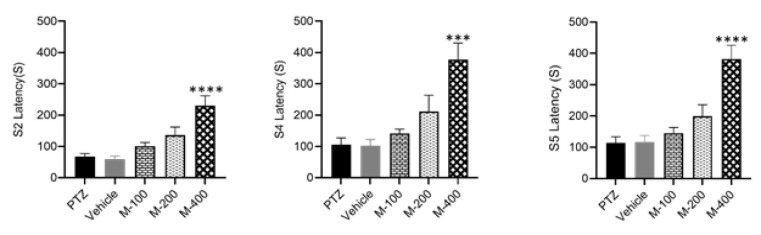
*Comparative analysis of latency (S2L, S4L, and S5L) for seizure stages in the experimental groups. One way ANOVA followed by Tukey’s post-test verified that 400 mg/kg of *P. lentiscus L. gum significantly increased the latency to reach seizure stages 2, 4, and 5 when compared with the PTZ group. Data are presented as mean±SEM; **p<0.01, ***p<0.001 and ****p<0.0001 compared to PTZ. S2L: stage2, S4L: stage 4 and S5L: stage5.

**Figure 2:**
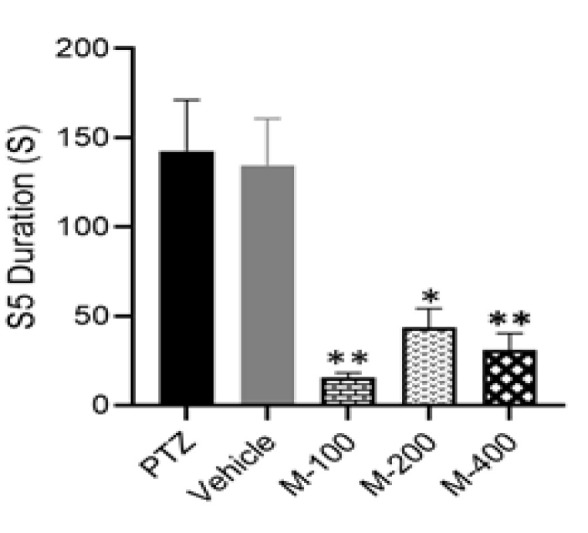
*Comparison of S5 duration (S5D) seizure stages in experimental groups. One way ANOVA followed by Tukey’s post-test verified that 400 mg/kg of *P. lentiscus *L.* gum significantly decreased the duration of stage 5 (S5D) when compared to the PTZ group. Data are presented as mean±SEM; *p<0.05 and**p<0.01 in comparison with the PTZ. S5D: stage 5 duration.

 One-way ANOVA, accompanied by Tukey’s post-test, was employed to compare the mean values of latency for reaching seizure stages 2, 4, and 5, as well as the duration of stage 5 across the experimental groups. As illustrated in Figure 1, the administration of 400 mg/kg dose of *P. lentiscus* L. gum significantly prolonged the latency to reach stages 2 (p<0.0001), 4 (p<0.001), and 5 (p<0.0001) of seizure, and reduced the duration of stage 5 when evaluated with respect to the PTZ group (p<0.01, Figure 2).

### P. lentiscus L. gum improved PTZ-induced oxidative stress in hippocampal tissues

**Figure 3:**
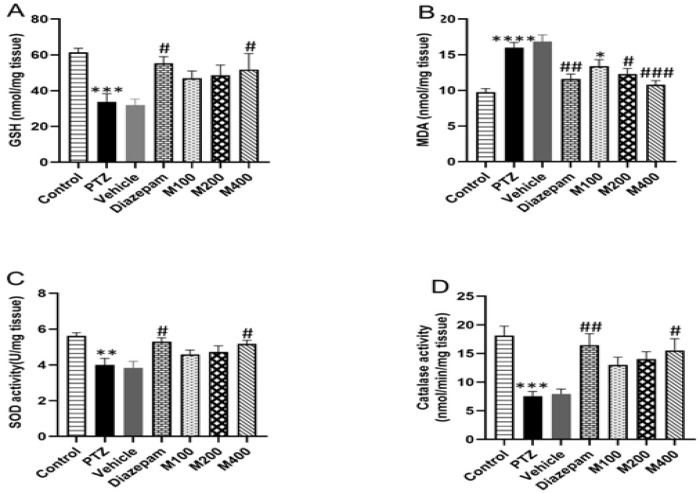
GSH content (A), MDA concentrations (B), and activities of SOD (C) and catalase (D) in the area of the hippocampus. MDA: Malondialdehyde; SOD: Superoxide dismutase; PTZ: Pentylenetetrazol. Data are presented as mean±SEM, n = 10. ***p<0.001, ****p<0.0001, and **p<0.01 versus the control group, #p<0. 05, ##p<0.01, and ###p<0.001 versus the PTZ group.

PTZ increased the concentration of MDA (16.01 ± 0.72) nmol/mg and decreased the GSH (33.85 ± 4.85) nmol/mg tissue, accompanied by CAT activity (7.53 ± 0.87, nmol/min/mg tissue), and SOD activity (4.01 ± 0.37, U/mg tissue) (p<0.0001, p<0.001 and p<0.001 and p<0.01 respectively, Figure 3). Additionally, treatment with *P. lentiscus *L. gum at doses of 200 and 400 mg/kg decreased the elevated MDA levels induced by PTZ (10.87 ± 0.59 and 12.3 ± 0.78, nmol/mg tissue respectively) in comparison to the PTZ (16.01 ± 0.72) group (p<0.001 and p<0.05, nmol/mg tissue respectively, Figure 3B). However, *P. lentiscus* L. gum at the dose of 400 mg/kg increased GSH (51.84± 3.65, nmol/mg tissue) when compared to the PTZ group (33.85 ± 4.85, nmol/mg tissue, p<0.05, Figure 3A). Further, 400 mg/kg of *P. lentiscus* L. gum increased SOD activity (5.18 ± 0.2, U/mg tissue) compared with the PTZ (4.01 ± 0.37, U/mg tissue) group (p<0.05, Figure 3C). Moreover, CAT activity (15.56 ± 2.03, nmol/mg tissue) in the *P. lentiscus* L. gum 400 mg group increased (p<0.05, Figure 3D) compared to the PTZ group (7.53 ± 0.87, nmol/mg tissue).

## Discussion

The anticonvulsant effects of *P. lentiscus *L. gum were evaluated using the PTZ-induced acute seizure. Furthermore, the antioxidant effects of *P. lentiscus* L. gum on hippocampal oxidative stress have been examined in this model. The findings suggest that a dosage of 400 mg/kg of *P. lentiscus* L. gum was effective in delaying the onset of seizure stages 4 and 5, which are associated with tonic-clonic seizures. Additionally, the duration of the fifth seizure stage was notably reduced across all administered doses. PTZ is recognized as an inhibitor of the gamma-aminobutyric acid (GABA-A) receptor, acting by targeting the picrotoxin region of this receptor, which increases the CNS neurons’ excitability. Thus, compounds that can diminish increased excitability or demonstrate inhibitory effects may serve as anticonvulsant and anti-epileptic agents in this model (Schmidt and Schachter 2014). A prior study indicated that the essential oil extracted from the resin of *P. lentiscus* L. gum predominantly contains compounds like α-pinene, β-pinene, and 4-carvomenthol. Researchers found that the α-pinene and 4-carvomenthol have strong binding affinity with GABA-A receptors and sodium channels, respectively (Jain et al. 2015). Furthermore, prior studies have indicated that 4-carvomenthol possesses anticonvulsant properties, while derivatives of α-pinene and β-pinene exhibit neuroprotective effects (Mechoulam et al. 1995; Sousa et al. 2009). These findings highlight the diverse pharmacological actions of the compounds present in Mastaki and their potential therapeutic benefits in the context of epilepsy and neuroprotection. Consistent with this information, a study has documented that the hydroalcoholic extract of another species of this plant, *Pistacia vera*, demonstrates muscle relaxant, and anti-anxiety properties (Ziaee and Hosseinzadeh 2010). These effects are believed to be linked to the augmentation of GABAergic neurotransmission. This suggests that compounds present in *Pistacia vera* may modulate GABA receptors, leading to the observed sedative, muscle-relaxing and anxiolytic effects (Ziaee and Hosseinzadeh 2010). Moreover, the contribution of oxidative stress to the pathophysiological mechanisms of epilepsy is broadly recognized. Research has indicated that during PTZ-induced epilepsy, there is an elevation in brain lipid peroxidation while the activity of antioxidant enzymes decreases. Furthermore, GABA-A receptors are particularly sensitive to free radicals and oxidative stress which have the potential to impede neurotransmission through these receptors. Additionally, free radicals can directly trigger epilepsy by deactivating glutamate decarboxylase, leading to the accumulation of glutamate in the brain. These mechanisms underscore the intricate interplay between oxidative stress, neurotransmission, and the progression of epilepsy (Aguiar et al. 2012). Consistent with the existing evidence, the current study revealed elevated levels of MDA and reduced glutathione levels, along with a decrease in the activity of antioxidant enzymes such as SOD and CAT in the hippocampus of rats subjected to PTZ administration. These findings align with previous research by Patsoukis et al., which proved that PTZ-induced seizures lead to oxidative stress specifically in the hippocampus. The observed changes in oxidative markers and antioxidant enzyme activities further support the notion that oxidative stress significantly contributes to the pathophysiology of epilepsy (Patsoukis et al. 2004). Additionally, another study has reported a reduction in the activity of antioxidant enzymes and an elevation in MDA levels in the rat hippocampus following PTZ administration (Zhu et al. 2015). Conversely, the antioxidant properties of phenolic and flavonoid components play a vital role in combating oxidative stress and protecting neuronal cells from damage associated with seizures.

Furthermore, it has been shown that oxidative stress might also contribute to a decrease in GABA-A receptor activity, which in turn, can increase neural activity and the risk of seizures (Accardi et al. 2014). Therefore, the use of herbal compounds rich in these antioxidants represents a promising approach to manage seizures and associated oxidative stress in the context of epilepsy (Gür et al. 2018). Recent research finding indicated that *P. lentiscus* L. contains abundant components with diverse antioxidant characteristics, including phenolic and flavonoid acids such as naringenin, eriodyctiol, daidzein, genistein, quercetin, kaempferol, apigenin, and luteolin (Elez Garofulić et al. 2020). Flavonoids exert their protective effects by stimulating the activity of antioxidant enzymes, scavenging free radicals, and inhibiting the activity of oxidizing enzymes. Specifically, hydroxyl flavonoids within this group can interact with free radicals, thereby shielding biological molecules from oxidative damage (Heim et al. 2002). Additionally, studies have shown that these components provide protective effects in animal seizure models. For instance, Han et al. showed that apigenin had beneficial effects on epilepsy through its antioxidant properties (Bozorgi et al. 2013; Han et al. 2012). Hence, based on the findings and evidence provided, it can be inferred that *P. lentiscus* L. gum alleviates the symptoms of PTZ-induced seizure by modulating GABAergic neurotransmission and exerting antioxidant effects.

*Pistacia lentiscus* L. effectively decreases seizure activity in PTZ-induced seizures in rats. This anticonvulsant effect is likely linked to its antioxidant properties. These results suggest that *Pistacia lentiscus* L. could be a valuable therapeutic option for epilepsy management.

## Data Availability

Data can be obtained upon request from the corresponding author.
